# Filling the Depths: Innovative Vertical Rectus Abdominis Myocutaneous (VRAM) Flap Reconstruction in a Complex Deep Tissue Pressure Injury

**DOI:** 10.7759/cureus.81302

**Published:** 2025-03-27

**Authors:** Amber R Jacobson, Jonathan Sarik, Hamdan Mallick, Lynnsey M Rebner, Annika Surapaneni

**Affiliations:** 1 General Surgery, Bayhealth Medical Center, Dover, USA; 2 Plastic Surgery, Bayhealth Medical Center, Dover, USA; 3 Medicine, Bayhealth Medical Center, Dover, USA; 4 Medicine, Drexel University College of Medicine, Philadelphia, USA

**Keywords:** deep tissue injury (dti), fascio-cutaneous flap, surgical technique wound closure, vertical rectus abdominis myocutaneous (vram) flap, wound repair

## Abstract

Deep tissue injuries (DTIs) pose significant reconstructive challenges, particularly when involving extensive soft tissue loss and exposure of vital structures. We report a case of a 69-year-old male with multiple comorbidities who developed a complex, full-thickness trochanteric wound following prolonged immobilization after a cardiac event. The injury, characterized by a 19 x 9 cm defect with compromised surrounding tissues - including the tensor fascia - precluded the use of conventional local flaps. Given the size and depth of the defect, a pedicled vertical rectus abdominis myocutaneous (VRAM) flap was selected for its robust vascularity, versatility, and ability to provide ample soft tissue bulk. Complete healing was observed at the six-month follow-up visit with no evidence of abdominal wall compromise or functional deficit. This case illustrates that the VRAM flap is a reliable and effective option for the reconstruction of large, complex DTIs in the proximal lower extremity, particularly when local flap options are compromised.

## Introduction

Deep tissue injuries (DTIs) represent a significant burden for patients and a challenge for reconstructive surgeons. These wounds are often chronic in nature; however, they can also develop acutely at sites of intense focal pressure. According to the National Pressure Ulcer Advisory Panel (NPUAP), a pressure ulcer is defined as a localized injury to the skin and/or underlying tissue usually over a bony prominence, as a result of pressure, or pressure in combination with shear and/or friction forces. Immobile patients, such as stroke, spinal cord injury, or unconscious patients paired with poor nutrition, are at risk for these wounds [1]. An estimated one to two million people per year develop pressure ulcers, with an estimated 60,000 mortalities each year from pressure ulcer complications [2]. A patient's nutritional status also plays a critical role in the development of pressure ulcers as well as healing [3].

Regardless of the cause, the wounds can be extensive with significant undermining and exposure of vital structures. Furthermore, these wounds are often complicated by the development of osteomyelitis, which is notoriously difficult to treat. Common sites of DTI include the sacrum, ischium, and trochanter as well as other bony prominences [4]. Reconstructive surgeons employ a variety of techniques to achieve stable wound coverage for DTI, such as large myocutaneous or fasciocutaneous advancement flaps, which are favored for sizable defects as they provide thick, well-vascularized tissue and can be harvested from a variety of locations. Most commonly, flaps are taken from nearby anatomical locations that can be translated tension-free into the defect. For trochanteric pressure injuries, the workhorse flap is commonly based upon the tensor fascia lata (TFL); however, other options include the gluteus maximus, gracilis, hamstrings, omentum, or perforator-based flaps such as the anterior lateral thigh and the profunda femorus artery [5,6]. When these local flaps are not a viable option due to an extensive or unusual wound, more remote sources of stable soft tissue coverage are sought. Here, we described a patient with an acute DTI involving the trochanteric region secondary to a prolonged period on the floor of his home after a cardiac event who ultimately was reconstructed using a pedicled vertical rectus abdominus myocutaneous (VRAM) flap to fill the defect.

## Case presentation

The patient is a 69-year-old male with a past medical history of atrial fibrillation, hypertension, stage III colon cancer, chronic obstructive pulmonary disease, peripheral vascular disease, obstructive sleep apnea, chronic kidney disease stage III, and hemochromatosis who had presented to an outside hospital for a syncopal episode secondary to a cardiac event. The patient was found down for at least 48 hours before caregivers found him.

**Figure 1:**
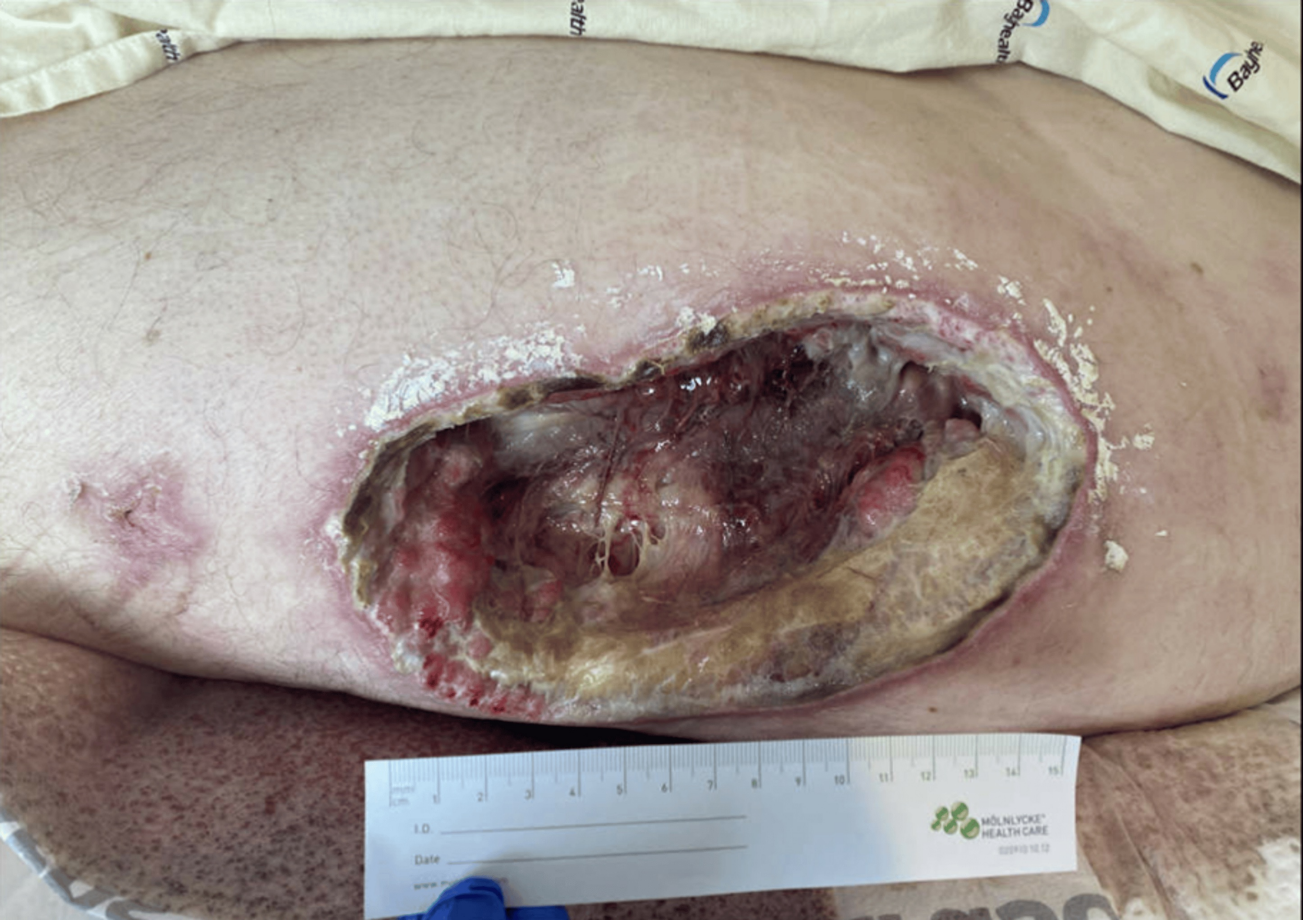
Left lateral thigh wound at initial consult showing over greater trochanter - 19 cm x 9 cm x 4.8 cm, stage 4

His level of care was quickly escalated to the ICU due to infected pressure injuries, as well as acute respiratory failure necessitating intubation and mechanical ventilation, likely secondary to pneumonia and rhabdomyolysis. The patient received serial wound debridement throughout his hospital stay and was eventually discharged to an acute rehab facility. There, they noted persistent drainage and poor wound healing, and the patient was sent to our emergency department for evaluation. He was then referred to plastic and reconstructive surgery for debridement and reconstruction of multiple pressure wounds involving his left greater trochanter (Figure 1) and left posterior shoulder.

**Figure 2:**
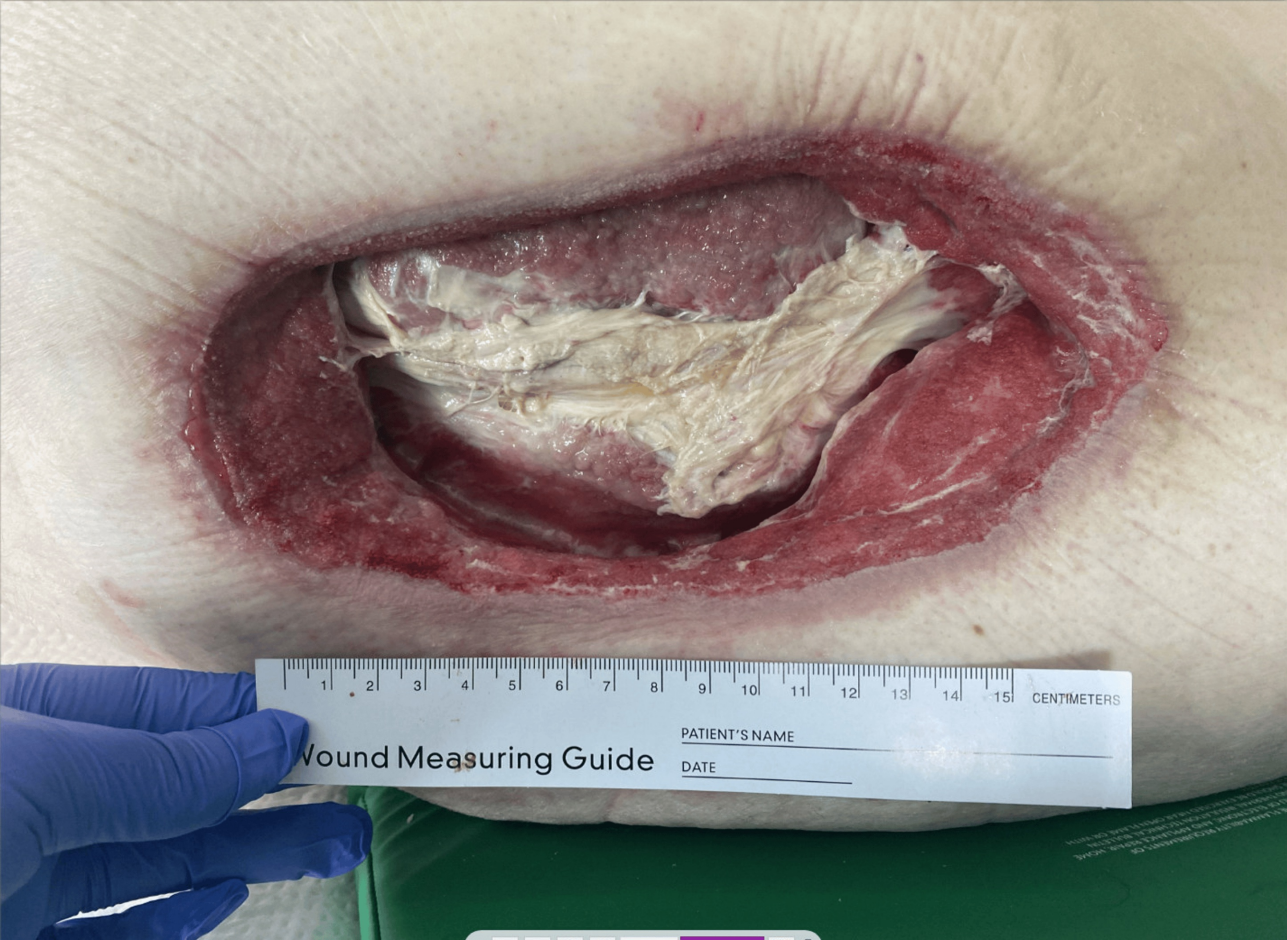
Left lateral thigh wound after debridement and wound vacuum therapy Left lateral thigh wound 3.5 weeks after initial debridement with interval wound vacuum therapy.

The patient initially had an albumin level of 1.8 g/dL and a hemoglobin of 9.8 g/dL, indicating some degree of baseline malnutrition. After appropriate medical optimization, he was then taken to the operating room for wound debridement and wound vacuum placement. Wound vacuum changes were done on a Monday-Wednesday-Friday basis. Deep tissue cultures from this debridement yielded a polymicrobial infection, including *E. coli* and *M. morganii*, and he was followed by infectious disease and placed on appropriate antibiotics based on culture sensitivities. Figure 2 shows the wound after initial treatment.

He was then brought back to the operating room three weeks after his initial debridement for final closure of his wounds. The left posterior shoulder wound, measuring 10 cm x 9 cm and involving the latissimus dorsi muscle, was closed with a fasciocutaneous advancement flap.

**Figure 3:**
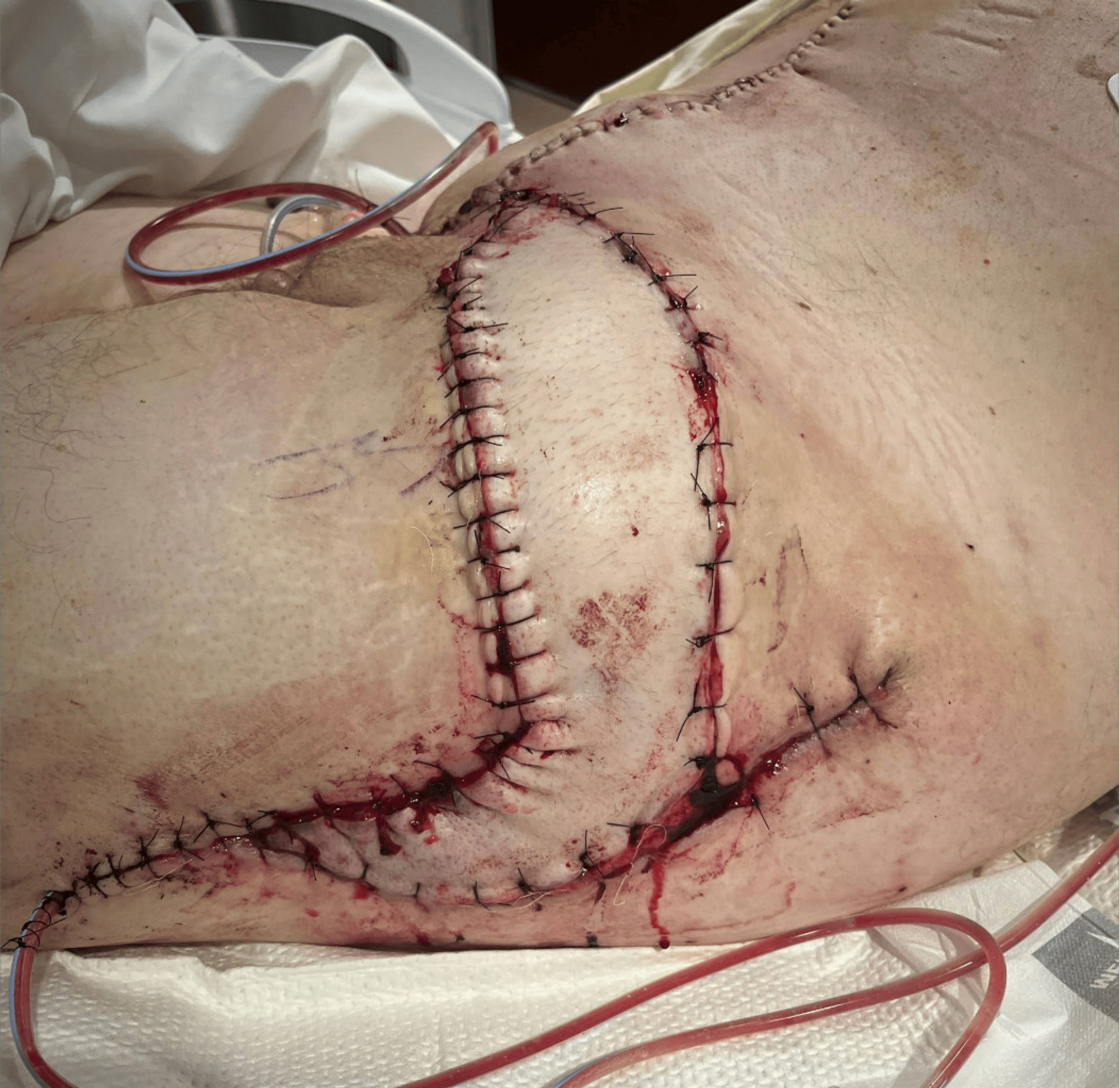
Flap of the left lateral thigh on postoperative day 1

**Figure 4:**
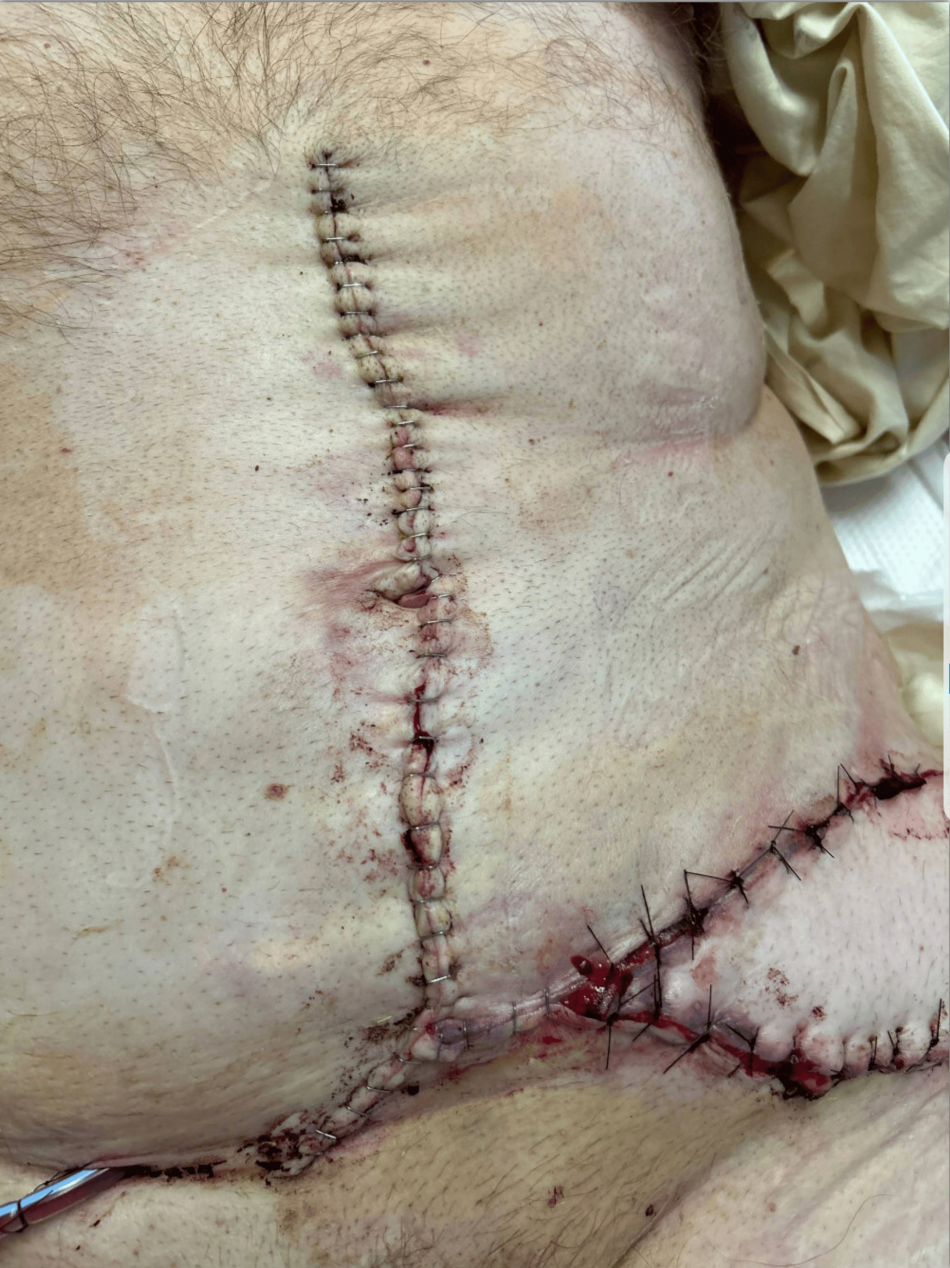
Donor midline abdominal wall incision, source of flap

The left greater trochanter injury was further debrided and was found to be a full-thickness defect involving the TFL and quadriceps muscle of the thigh. Given that the defect measured 19 cm x 9 cm, primary closure and adjacent tissue transfers were deemed inadequate to achieve stable wound coverage; thus, we proceeded with the planned VRAM reconstruction. A template for the defect was measured, designed, and translated onto the abdomen to plan for the necessary skin island. The flap was raised fully after carefully skeletonizing the inferior epigastric pedicle. Care was taken to preserve as many perforating vessels as possible to fully supply the large skin island. The flap was then rotated to the left hip and found to have appropriate length for the pedicle as well as an excellent size match for the defect. A skin bridge was then incised from the left lower quadrant of the abdomen to the defect to allow for tension-free transposition of the flap as well as to accommodate the expected edema around the pedicle. The abdomen was closed with Prolene mesh. Drains were also placed into the donor site as well as beneath the flap. Figure 3 shows the flap on postoperative day 1, and Figure 4 shows the midline abdominal donor site.

**Figure 5:**
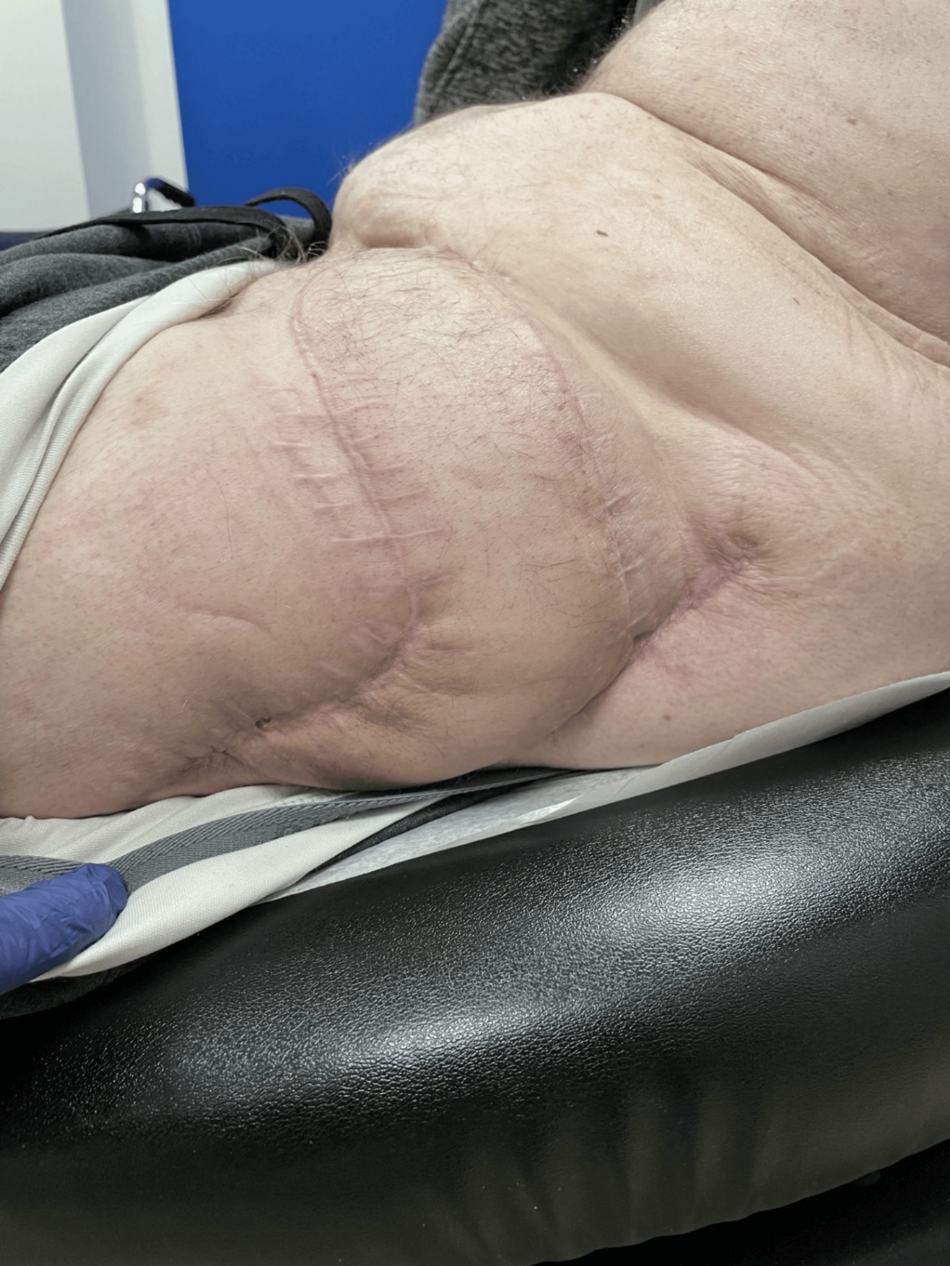
Healed left lateral thigh flap six months postop

**Figure 6:**
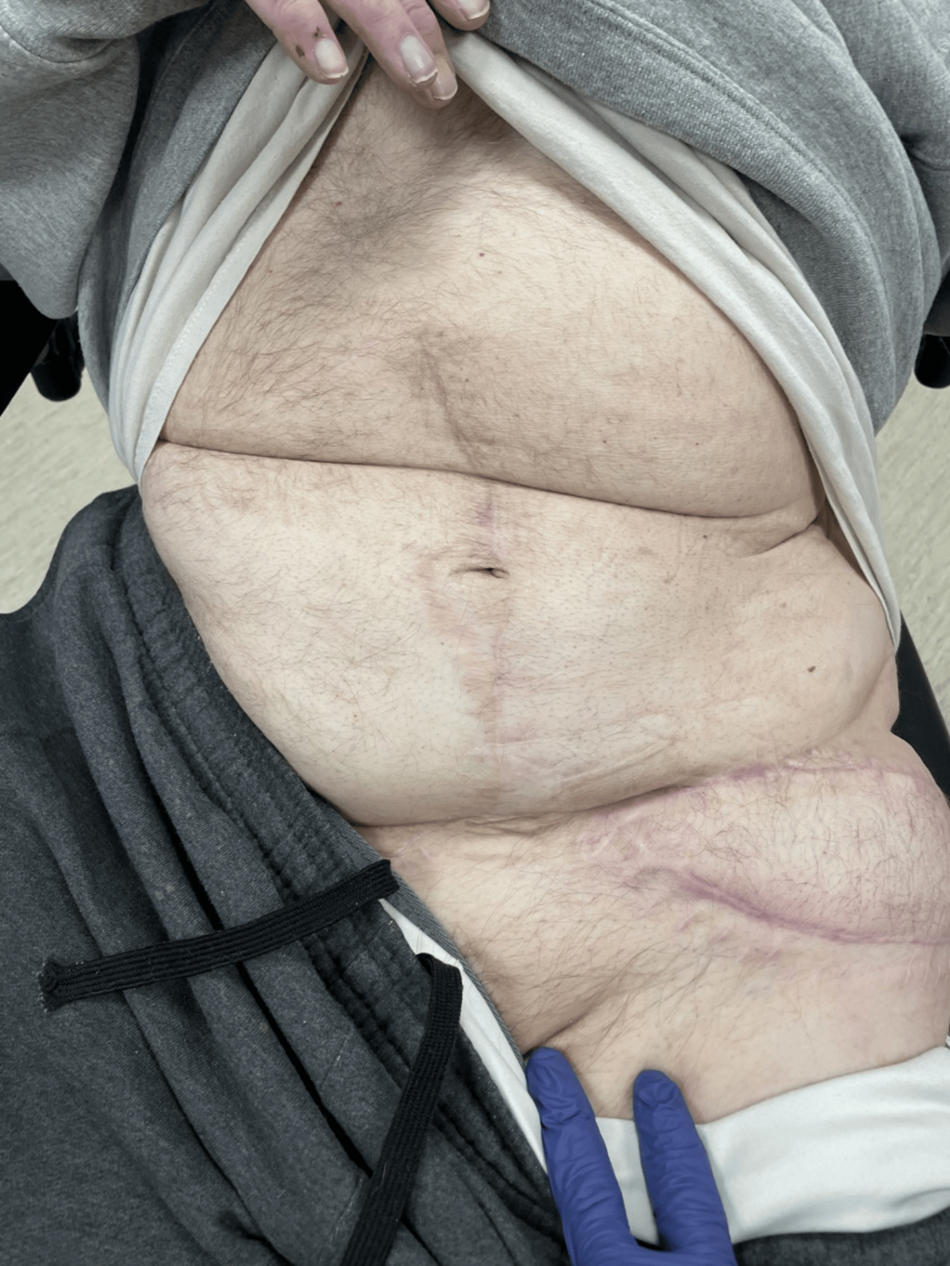
Healed midline abdominal donor site six months postop

The patient followed our postoperative recovery pathway and was kept on strict bedrest for four days, followed by intermittent periods of ambulation until he was advanced to weight bearing as tolerated. Due to his ongoing recovery from his cardiac event, as well as the additional needs for his reconstruction, he was eventually discharged to a skilled nursing facility. His initial postoperative course was uncomplicated except a small area of delayed healing at the inferior aspect of the abdominal donor site, which was managed with additional negative pressure wound vacuum therapy. This eventually resolved without further intervention, and the wound vacuum was discontinued. The remainder of his recovery was uneventful, and he continued to work with physical therapy to regain strength and progress towards his prehospital state. At the six-month visit, he had been discharged to home care with physical therapy and demonstrated complete healing of both the donor site as well as his reconstruction. Physical exam was negative for any discernable bulge to his abdominal wall or functional deficits related to his donor site. Figure 5 shows the healed flap six months post-surgery, and Figure 6 shows the midline donor site.

## Discussion

The case presented describes the reconstruction of an acute DTI to the left lateral thigh area using an abdominal VRAM flap. As with many pressure ulcers, the sacral, ischial, and trochanteric regions are particularly vulnerable due to the presence of large bony prominences underlying soft tissue. This pressure along the bone causes an initial point of emaciation, and continued pressure further exacerbates tissue damage, resulting in deepening necrosis. The margins of the wound then roll into itself, further deepening the ulcer pocket. Without adequate intervention, the wound can extend to involve deeper structures such as bone, increasing the risk of complications like osteomyelitis and septic arthritis [4]. Patients who are nutritionally deprived are at risk for these wounds escalating in stage, with an albumin level of < 3.1 g/dL being a predictor of pressure wound formation along with associated higher mortality [2].

Our patient, found immobile in the left lateral decubitus position for several days, developed ischemia on his left trochanteric and left scapular prominence, leading to extensive necrosis. His pre-op and post-op albumin levels indicated chronic malnutrition with a consistent albumin of < 3 g/dL. Pre-operative management began with nutritional optimization and multiple debridement of necrotic tissue, aligning with standard practice as described by Daneshgaran et al. [7]. Additionally, MRI imaging was performed before reconstruction to exclude osteomyelitis. Given the severity and depth of the patient’s wound, along with his nutritional status, a musculocutaneous flap with a robust blood supply was necessary to ensure durable coverage and healing.

In a similar case report, a patient with a large trochanteric injury used three local flaps (TFL and two fasciocutaneous flaps) to cover the defect [8]. While regional flaps such as the TFL are often the first line of consideration for trochanteric wounds, this was not feasible for this patient as the TFL was compromised by the wound itself, along with the wound's size and depth [5,6]. Free perforator flaps have also been used in trochanteric defects. In a study of 10 patients, multiple different types of perforators were used successfully [9]. Similarly, Koenig et al. described a successful case of vascularized nerve graft and fascial free flap in lower extremity reconstruction, further illustrating the feasibility of microvascular approaches in challenging defects [10]. For our patient, however, we determined that this option would not provide sufficient coverage or depth for his defect. Moreover, the prolonged operative times required for free tissue transfer made it less favorable given his multiple comorbidities and recent cardiac event.

The VRAM flap, on the other hand, provided several key advantages: it is highly versatile - allowing for various orientations (extended, oblique, or vertical) of the vascular pedicle, has a long arc of rotation, and contains excellent vascularity. It is classified as a type III muscle flap, meaning that it is supplied by two large vascular pedicles from separate sources (the inferior and superior epigastric arteries). This increases the muscle's survivability even if one pedicle is divided during flap elevation, as was done for this patient. Furthermore, this design permits partial muscle usage to further fill the deep space of the trochanteric wound [4]. VRAM flaps have been most commonly used in nearby reconstruction, with case reports showcasing its use in suprapubic wounds and even above the knee amputation stumps [11,12].

Despite the limited number of cases in which the VRAM flap has been utilized for trochanteric pressure injuries, there are a few studies on the outcomes of proximal thigh and groin reconstruction. A retrospective cohort and systematic review of VRAM flaps for proximal thigh and groin reconstruction found that out of 53 cases, the success rate was high, with failure rates reported at only 1.9%, with minimal rates of necrosis and infection [13]. Another study looked at the outcomes for a similar group of patients with proximal thigh and groin defects that were irradiated for cancerous lesions. Out of the 50 patients, all flaps survived with only three patients requiring additional coverage procedures due to partial necrosis [14]. These robust success rates in both studies further support the VRAM flap use in complex wounds such as our patient.

Another known potential complication associated with the VRAM flap, unlike fasciocutaneous flaps, is the risk of abdominal wall hernia with a 15.6% incidence [13]. Despite this concern, the overall benefit of achieving successful wound coverage outweighed this risk in the present case, particularly since the patient had no history of abdominal wall hernias or defects, however, it still remains an important consideration. This risk was also minimized by placing a Prolene mesh just superficial to the posterior fascia. The patient's donor site at the six-month postoperative visit healed well without any signs of bulging or hernias on examination.

## Conclusions

The VRAM flap is a viable option for reconstruction for DTIs of the trunk and proximal lower extremities, offering a robust vascular supply, directional versatility, and reliable wound coverage in scenarios where alternative flaps were either compromised or unsuitable for adequate coverage. Following his recent cardiac event along with his poor nutritional status, the patient was unable to endure the extended operative times required for a free tissue transfer, and the depth of his wound also precluded the use of local advancement flaps such as the TFL. These factors made him an ideal candidate for VRAM flap reconstruction. However, VRAM flap reconstruction has its limitations, including donor site complications in patients with poor nutritional status or when free flap transfer is preferred for more distant defects. Following proper medical optimization and appropriate rehabilitation, the patient's flap healed appropriately, and he has since returned to his functional baseline status. 
